# Effect of long-term adjuvant temozolomide chemotherapy on primary glioblastoma patient survival

**DOI:** 10.1186/s12883-021-02461-9

**Published:** 2021-11-02

**Authors:** Bin Huang, Zuan Yu, Risheng Liang

**Affiliations:** Department of Neurosurgery, Fujian Medical University Union Hospital, No. 29 Xinquan Road, Gulou District, Fuzhou City, Fujian Province China

**Keywords:** Glioblastoma, Long-term adjuvant chemotherapy;progression-freesurvival, Cancer survival

## Abstract

**Objective:**

Glioblastoma multiforme (GBM) is the most common primary malignant central nervous system (CNS) tumor. The Stupp regimen is the standard treatment, although the optimal number of temozolomide (TMZ) treatment cycles remains controversial. We compared the effects of standard 6 cycles versus > 6 cycles of TMZ chemotherapy post-surgery with concurrent chemoradiotherapy on primary GBM patient survival.

**Patients and methods:**

We performed a single center retrospective study of GBM patients that underwent total resection, concurrent chemoradiotherapy, and at least 6 cycles of adjuvant TMZ chemotherapy from June 2011 to August 2018. Patients were divided into 2 groups based on adjuvant TMZ treatment plan: Group A(*n* = 27): standard 6-cycle adjuvant TMZ therapy and Group B(*n* = 26): > 6 cycles of adjuvant TMZ therapy. Primary endpoints were progression-free survival (PFS) and overall survival (OS). Continuous variables were analyzed by ANOVA, and the Kaplan-Meier method was used to evaluate PFS and OS. Univariate and multivariate COX analyses determined correlation between survival rates and covariates. We used The Mini Mental State Examination (MMSE) and Karnofsky Performance Status (KPS) to assess patients’ neurocognitive function and quality of life.

**Results:**

After follow-up, median PFS was 15 months in in Group A (95%CI 9.5–20.5) and 20.1 months in Group B (95%CI 15.9–24.4). Group A median OS was 19.4 months (95%CI 15.5–23.2), compared to 25.6 months in Group B (95%CI 20.4–30.8). The 2-year survival rate of Groups A and B was 36% was 66%, respectively (*P* = 0.02). and 5-year survival was 7% in both. Multivariate COX regression analysis showed association between patient PFS and long-period adjuvant chemotherapy, but not OS. There were no significant difference in disability or quality of life during treatment with Stupp protocol, but differences in MMSE and KPS were in favour of the Groups B after year 1 of the treatment (*P* < 0.05).

**Conclusions:**

Long-term adjuvant TMZ chemotherapy was beneficial for PFS and 2-year survival rate in GBM patients, and improved their quality of life contemporarily. But OS was not significantly improved.

## Introduction

Glioblastoma mulitforme (GBM) is the most common primary malignant tumor of the adult central nervous system (CNS), accounting for 45.2% of all CNS malignancies and an annual incidence of 3 out of 100,000 [1]. After the phase III clinical trial conducted by Stupp et al., the standard treatment for newly diagnosed GBM is post-surgical radiotherapy (RT) or biopsy and 75 mg/m^2^ daily adjuvant therapy with temozolomide (TMZ). TMZ is a common orally administered chemotherapeutic compound that acts via guanine methylation and subsequent inhibition of cellular proliferation. Six cycles of adjuvant TMZ therapy were administered performed following radiotherapy (28 days per cycle, with TMZ given the first 5 days per cycle). However, despite standard Stupp treatment, the prognosis of most GBM patients remains poor, with a median survival time of 14.6 months, a 26.5% 2-year survival rate, and < 5% five-year survival rate [2].

Weller et al. indicated that favorable prognostic factors for GBM include age, preoperative KPS score, IDH1/2 mutation, and levels of methylguanine-DNA methyltransferase (MGMT) promoter methylation [3]. The MGMT is recognized as a biomarker, as well as a primary contributor to TMZ resistance in glioblastoma [4]. Long-term TMZ administration will minimize MGMT levels and weaken tumor cell resistance, thereby “autonomously” enhancing anti-tumor effects of TMZ [5]. However, it remains debatable what is considered the optimal number of adjuvant TMZ therapy cycles [6].

Primary adverse reactions of adjuvant TMZ chemotherapy include thrombocytopenia and neutropenia, though studies suggest that long-term or high-dose adjuvant TMZ therapy does not increase the probability of neutropenia and thrombocytopenia compared to standard 6-cycle chemotherapy [7, 8]. Therefore, it is an appealing option for patients that have successfully completed 6 cycles of adjuvant chemotherapy [9]. With regard to these points of consideration, the goal of the present study was to evaluate whether long-term adjuvant TMZ chemotherapy could confer clinical benefits.

## Methods

### Patient information

The study was approved by Ethics Committee of Union Hospital of Fujian Medical University and performed according to the Declaration of Helsinki guidelines. Informed consent was obtained from the participants. In this study, included patients were from the Union Hospital of Fujian Medical University. Our hospital has conducted MGMT assessment since 2011 and TMZ has been a first-line medication for patients with GBM. Therefore, patients initially diagnosed with GBM (based on the WHO 2007 Central Nervous System Tumor Classification) between 2011 and 2018 were included in our study [10].

Specific inclusion and exclusion criteria were defined as follows. Patients had to be adults (≥18 years old and ≤ 70 years old) with histologically diagnosed WHO grade IV GBM. All included patients underwent total resection of the tumor in our Neurosurgery Department. Total resection is defined as complete tumor resection as determined via a T1-weighted MRI enhancement image of a postoperative brain [11]. All patients exhibited tumors located in non-critically functional regions of the cerebrum. The median time of radiation therapy (RT) was 4 weeks (3–6 weeks) surgery with adjuvant TMZ treatment. Later, TMZ was the first-line adjuvant chemotherapy for at least 6 treatment cycles. Patients in transition from low-grade glioma to GBM were excluded from the study. As the study objective was to evaluate potential adjuvant therapeutic benefits over 6 or more treatment cycles, patients that failed to complete the Stupp treatment program at our hospital were excluded, including those with tumor recurrence during 6-cycle adjuvant chemotherapy and more than 6 weeks of post-surgical radiotherapy [12]. Additional eligibility criteria included a pre-operative minimum KPS score of 60 and follow-up MRI every 3 months. All patient data were reviewed by an experienced neurosurgeon, including clinical evaluations, pathology report, and all imaging results.

We collected all data from electronic medical records, which included age, gender, the number of cerebral lobes involving tumors, and molecular markers (IDH mutation, MGMT methylation). A record of treatment process and procedures was obtained, including radiation dose, range, and number of adjuvant TMZ therapy cycles. The date of diagnosis was defined as the date of GBM diagnosis through histology. Time to first relapse was determined by histological examination (including surgical resection and biopsy) or through follow-up imaging data evaluation. Overall survival (OS) was defined as the timeframe between initial diagnosis and date of death or last follow-up. We used the MMSE and KPS scoring scales to collect survival quality from preoperative(T1), 7 days after surgery (T2), at the completion of the STUPP protocol (T3), and 1 year after surgery (T4), respectively.

### Statistical analysis

All data were recorded using Microsoft Excel (2007), and statistical analyses were performed using SPSS statistical analysis software (version 21.0). Statistics were plotted using GraphPad Prism software (GraphPad, Inc.). The Kaplan-Meier method was used for univariate survival analysis to estimate patient PFS and OS probability distributions following treatment. Cox regression models were used to analyze relationships between survival and covariates. The effect of each covariate on GBM treatment was gradually determined by selecting and analyzing different covariates.

## Results

Our initial search of the medical database identified 319 patients with GBM as the primary diagnosis, of which 252 were excluded for failing to meet the Stupp therapy criteria or due to receipt of follow-up treatment at other institutions. Of the remaining 67 patients, 8 were excluded as they less than 18 years of age (3patients) older than 70 years of age (5 patients). Six were excluded due to diagnosis of secondary glioblastoma. The remaining 53 patients were grouped as described in Table 1. Included patients were divided into two groups: Group A and Group B. Group A (*n* = 27) completed the Stupp protocol, and Group B (*n* = 26) continued adjuvant TMZ chemotherapy following completion of the Stupp protocol until tumor progression or patients refused continuation of chemotherapy. All patients underwent total tumor resection and were examined by T1-enhanced weighted MRI within 1 week after surgery to confirm complete resection of the tumor. The median cycle of adjuvant TMZ chemotherapy in Group B patients was 10 (range = 7–41). The first cycle of adjuvant chemotherapy lasted 28 days, and all patients were given adjuvant chemotherapy of 150 mg/m^2^ temozolomide for 5 consecutive days. If no treatment-related adverse reactions were observed in subsequent cycles, the dose was increased to a 200 mg/m^2^ adjuvant TMZ chemotherapy regimen.

**Table 1:** Summary of patient characteristics

	Group A	Group B	***P***
	(n = 27)	(n = 26)	
Sex			
Male	16 (59%)	14 (53%)	
Female	11 (41%)	12 (47%)	0.74
Onset age			
< 45	16 (59%)	12 (46%)	
> 45	11 (41%)	14 (54%)	0.26
Pre-surgery KPS			
60–80	5 (18%)	6 (23%)	
> 80	22 (82%)	20 (77%)	0.42
Brain lobes involved in tumor			
1	14 (52%)	15 (57%)	
> 1	13 (48%)	11 (43%)	0.42
MGMT methylation			
Yes	12 (44%)	14 (54%)	
No	13 (48%)	10 (38%)	
Unknown	2 (8%)	2 (8%)	0.78
IDH mutation			
Mutated	5 (18.5%)	8 (31%)	
Wild type	17 (63%)	12 (46%)	
Unknown	5 (18.5%)	6 (23%)	0.14
TMZ therapeutic cycles			
6	27 (100%)	0	
7–9		10 (38%)	
10–12		9 (34%)	
> 12		7 (28%)	< 0.001
Total patient number	27	26	53

Upon completion of the follow-up on August 2019 (median follow-up of 26 weeks; range = 13–72 weeks), 42 progression events occurred (24 in 89% of Group A and 69% in Group B). Seven patients (26%) in Group A survived to follow-up and 20 patients (74%) died from tumor progression. Ten patients (38%) in Group B survived to follow-up and 16 (62%) died from tumor progression. The 2-year survival rate for group A was 36%, compared to 66% in Group B (*P* = 0.02). Two patients (7%) in each group survived beyond 5 years.

**Fig. 1:**
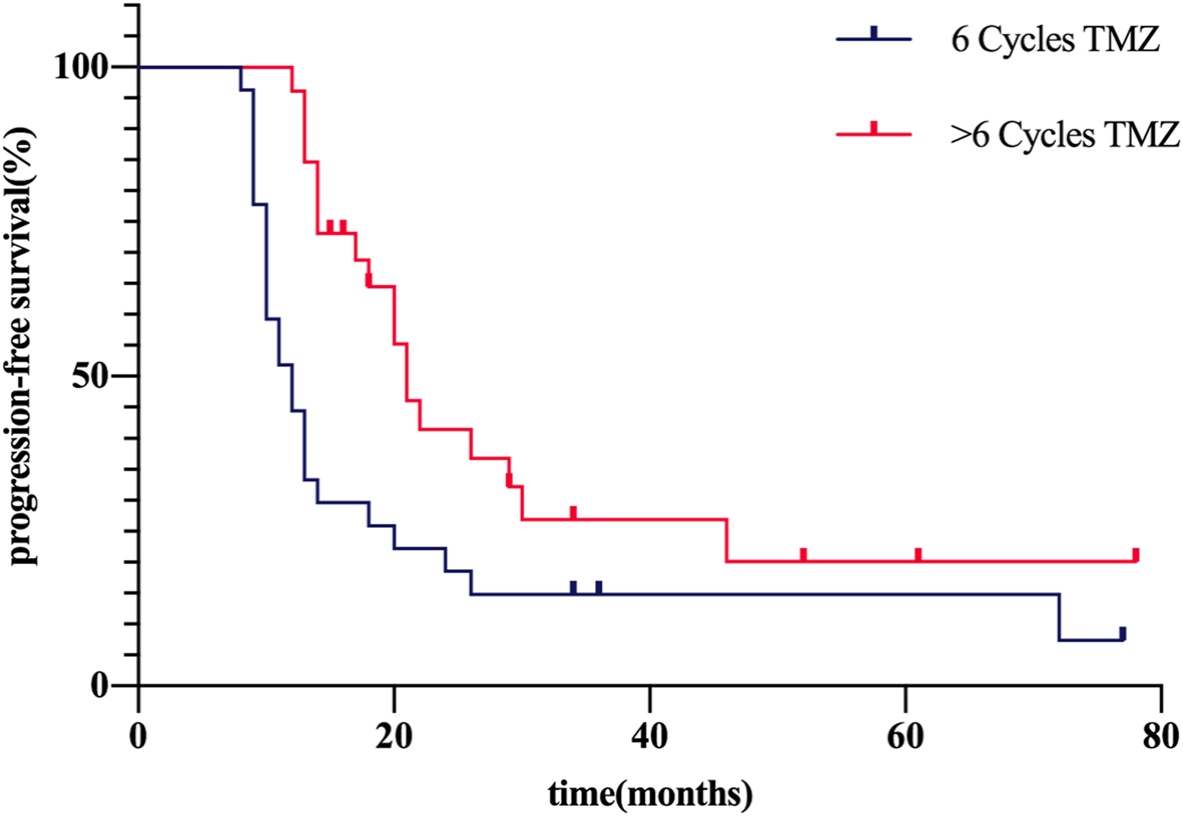
Progression-free survival curve for patients diagnosed with GBM after temozolomide adjuvant chemotherapy

**Fig. 2:**
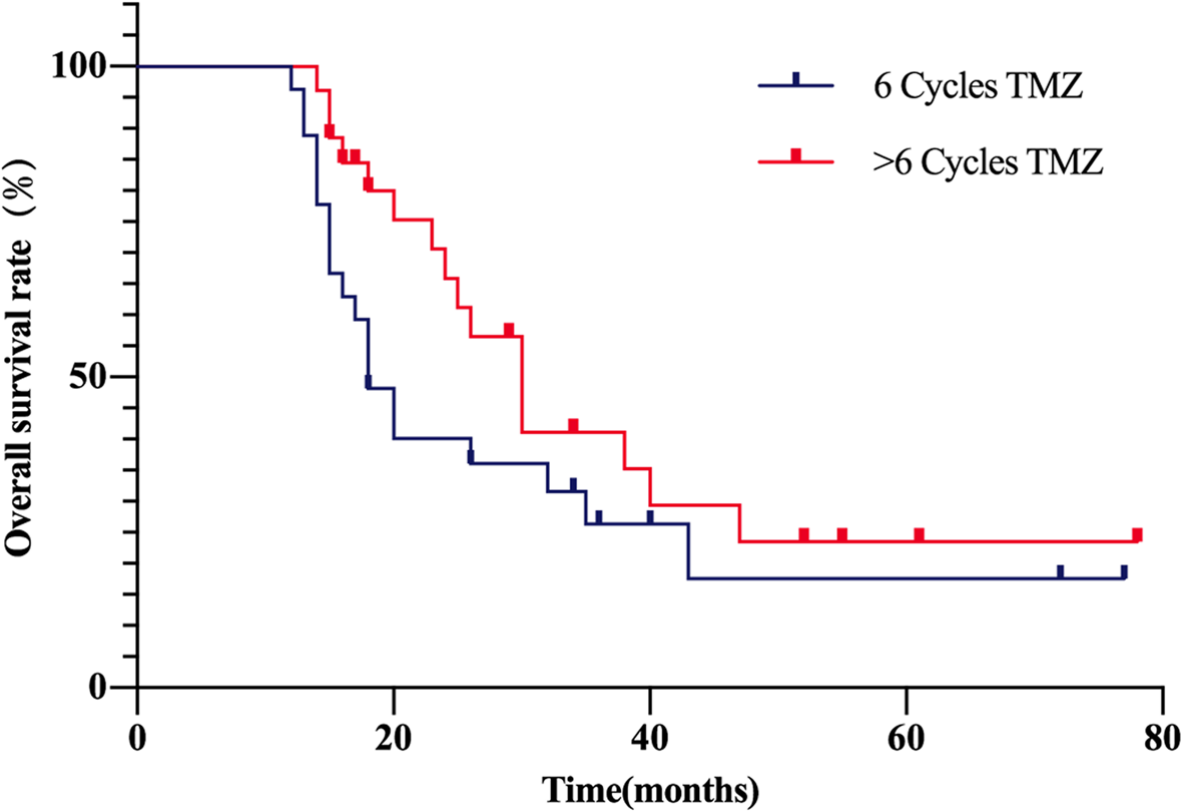
Overall survival curve for patients diagnosed with GBM after temozolomide adjuvant chemotherapy

The Kaplan-Meier method was used for comparing survival of standard and long-period adjuvant chemotherapy. The median progression-free survival (PFS) in Group A was 15 months (95% CI 9.5–20.5), compared to 20.1 months in Group B (95% CI 15.9–24.4). The median overall survival (OS) in Group A was 19.4 months (95% CI 15.5–23.2) compared to 25.6 months in Group B (95% CI 20.4–30.8). The Log rank method was used to test differences in survival time distribution. Patients that received long-term adjuvant chemotherapy exhibited a statistically significant PFS (χ2 = 7.06, *P* = 0.008, Fig. 1), but no difference was observed for OS time distribution (χ2 = 2.04, *P* = 0.152, Fig. 2).

Univariate analysis using the Cox proportional hazard model was performed (Table 2). Our results showed that the number of lobes involving the tumor, MGMT methylation, IDH mutation, and adjuvant TMZ chemotherapy cycles (HR:0.454; *P*:0.01) are factors related to tumor progression. Gender, age and pre-operative KPS score were not associated with tumor progression. OS was associated with the methylation status of MGMT, IDH mutation, pre-surgical KPS score, but not with age, gender, adjuvant chemotherapy cycles (HR:0.628; *P*:0.159) or the number of lobes with tumor involvement.

**Table 2:** Univariate analysis with regard to tumor progression or death

	Patient number	PFS	P值	OS	*P*
		HR(95%CI)		HR(95%CI)	
Age					
< 45	32	1		1	
> 45	21	1.227	0.511	1.341	0.384
		(0.667–2.258)		(0.692–2.597)	
Sex					
Female	23	1		1	
Male	30	1.062	0.843	1.19	0.586
		(0.587–1.920)		(0.629–2.269)	
Pre-surgery KPS					
< 80	11	1		1	
> 80	42	0.701	0.33	0.387	0.013
		(0.343–1.434)		(0.183–0.817)	
Brain lobes involved in tumor					
Single lobe	34	1		1	
Multiple lobes	19	0.521	0.04	0.783	0.365
		(0.278–0.975)		(0.382–1.424)	
MGMT methylation					
No	23	1		1	
Yes	26	0.343	< 0.001	0.379	0.005
		(0.178–0.663)		(0.192–0.748)	
IDH					
Wild-Type	29	1		1	
Mutation	10	0.222	0.01	0.112	< 0.001
		(0.682–0.07)		(0.027–0.56)	
TMZ therapeutic cycles					
6	27	1		1	
> 6	26	0.454	0.01	0.628	0.159
		(0.244–0.842)		(0.324–1.215)	

*Abbreviations*: *CI* Confidence interval, *KPS* Kanovsky performance score, *MGMT* O-6-methylguanine-DNA-methyltransferase, *IDH* Isocitrate dehydrogenase, *HR* Hazard ratio

When adjusted to a multivariate COX risk model with known variables (age, MGMT methylation, IDH mutation, adjuvant chemotherapy cycle number, KPS score, and number of lobe involvement), our analysis showed that PFS was associated with MGMT methylation (HR:0.336, *P*:0.002), IDH mutation, number of adjuvant TMZ chemotherapy cycles (HR:0.224, *P*:< 0.01) and the number of tumor-involved lobes. OS was associated with the methylation status of MGMT, IDH mutation and pre-operative KPS score, but not with the number of TMZ chemotherapy cycles or the number of involved cerebral lobes (Table 3).

**Table 3:** Multivariate analysis with regard to tumor progression or death

	Patient number	PFS	*P*	OS	*P*
		HR		HR	
		(95%CI)		(95%CI)	
MGMT methylation					
No	23	1		1	
Yes	26	0.336	0.002	0.470	0.025
		(0.167–0.674)		(0.243–0.909)	
IDH					
Wild type	29	1		1	
Mutation	10	0.094		0.077	
		(0.026–0.346)	< 0.001	(0.016–0.384)	0.002
TMZ therapeutic cycles					
6	27	1		–	
> 6	26	0.224	< 0.001	–	
		(0.106–0.473)			
Brain lobes involved in tumor					
Single lobe	34	1			
Multiple lobes	19	0.406	0.012	–	
		(0.201–0.819)			
Pre-surgery KPS score					
< 80	11	–		1	
> 80	42	–		0.192	< 0.001
				(0.079–0.464)	

*Abbreviations*: *CI* Confidence interval, *KPS* Kanovsky performance score, *MGMT* O-6-methylguanine-DNA-methyltransferase, *IDH* Isocitrate dehydrogenase, *HR* Hazard ratio

The median preoperative MMSE score of 21(range 12–29), a median preoperative KPS of 80/100. There were no significant difference at T1-T3 of *MMSE* scores and KPS scores between the two groups. Both patient group showed the highest KPS and MMSE at T3. After 1 year of treatment, the KPS score and MMSE score of patients in group B were higher group A, and the difference between the two groups was statistically significant (Tables 4 and 5).

**Table 4:** Longitudinal comparison of KPS scores in the two groups

KPS score	Group A(n = 27) Mean ± SD	Group B (n = 26) Mean ± SD	***P*** value
T1	79.62 ± 8.07	80.38 ± 12.15	0.792
T2	73.70 ± 6.87	73.46 ± 6.89	0.899
T3	91.11 ± 8.00	93.46 ± 6.89	0.257
T4	71.11 ± 21.89	89.61 ± 13.10	0.001

**Table 5:** Longitudinal comparison of MMSE scores in the two groups

MMSE score	GroupA(n = 27) Mean ± SD	Group B (n = 26) Mean ± SD	***P*** value
T1	21.59 ± 4.19	21.65 ± 4.56	0.960
T2	19.96 ± 4.55	19.53 ± 4.36	0.730
T3	24.77 ± 2.76	24.84 ± 2.98	0.931
T4	20.37 ± 5.83	23.84 ± 4.49	0.019

## Discussion

The European Organisation for the Research and Treatment of Cancer (EORTC) and the Canadian National Cancer Institute Clinical Trial Team (NCIC) conducted a phase III clinical trial in 2005 (NCT00006353) on the standard treatment protocol of Glioblastoma multiforme [2]. This trial established 6 cycles of adjuvant TMZ chemotherapy as the standard primary glioblastoma treatment protocol post-surgery and concurrent chemoradiotherapy (ie, Stupp protocol). Ultimately, 36.5% (105/287) of patients completed the full adjuvant chemotherapy course. However, there is no effective supplementary treatment after completing the Stupp program. Although six adjuvant TMZ therapy cycles are used in the Stupp regimen, the optimal regimen for such a therapy is debated. In clinical practice, some clinicians adopt a dose-intensive regimen or extend adjuvant treatment cycles beyond 6 weeks. Therefore, establishing a standardized adjuvant TMZ treatment plan is of high importance.

Prolonged exposure to alkylating agents will deplete intracellular MGMT in peripheral blood mononuclear cells, and low levels of MGMT will ensure optimal cytotoxicity of TMZ [4]. To verify whether lower MGMT levels were associated with improved survival, a phase III clinical trial divided patients with primary GBM into standard-dose and dose-dense TMZ treatment groups [8]. The results of this trial revealed a median OS of 16.6 and 14.9 months (HR 1.03; *P* = 0.63) and median PFS of 5.5 and 6.7 months (HR 0.87; *P* = 0.06), respectively, with no significant differences between the groups. Extended OS was not observed in the dose-dense group, although the incidence of adverse reactions was greater in this group compared to the standard-dose group (52.5 and 34.1%).

Few large long-term adjuvant chemotherapy studies are present in the published literature. In several retrospective studies [13–15], the median chemotherapy cycle number ranged from 14 to 16 cycles in long-term adjuvant chemotherapy groups. In these studies, patients undergoing long-term TMZ adjuvant chemotherapy treatment exhibited longer PFS and OS than those receiving standard chemotherapy regimens. Such studies indicate that the number of adjuvant TMZ chemotherapy cycles is an independent factor that benefits both PFS and OS; however, our findings suggest otherwise. Long-term adjuvant TMZ chemotherapy improved PFS (HR:0.454; *P*:0.01) not OS (HR:0.628; *P*:0.159) based on Cox regression and survival curve analyses in our study. Similar studies have been reported in the literature [16–18]. Gramatzki, D. et al. evaluated 142 newly diagnosed GBM patients between 2004 and 2010 [16]. The study determined that long-term adjuvant chemotherapy was independently associated with PFS, but COX regression did not support and benefit to OS. Skardelly et al. studied 107 recently diagnosed GBM patients from 2006 to 2014 [17]. In their study, long-period adjuvant chemotherapy group exhibited a higher median survival time than the standard-period adjuvant chemotherapy group (28.6 months and 25.2 months). However, following multivariate regression analysis, no significant differences between the two groups were determined (RR 0.77, *P* = 0.46).

Researchers have observed improvements in PFS from long-cycle chemotherapy on, as reported by multiple publications. Whether or not there is a statistically significant difference in OS, the long-cycle adjuvant TMZ chemotherapy group shows a higher 2-year survival rate [13–18]. In the present study, the 2-year survival rate of patients in the standard- and long-cycle adjuvant TMZ chemotherapy groups were 36 and 66%, respectively (*P* = 0.02). We believe that an increased 2-year survival rate in the long-term adjuvant chemotherapy group is associated with prolonged PFS from long-term chemotherapy. In a retrospective analysis of phase II clinical trials, 437 GBM patients included were divided into 9-week, 18-week, and 26-week groups according to post-operative PFS. The findings of this study show that patients with extended PFS also have higher survival rates after tumor recurrence [19]. A retrospective analysis of 831 GBM patients included in trial RTOG 0525 showed that the risk of death after GBM progression was 6.6 times higher than in the group that did not exhibit cancer progression [20]. There is a close correlation between PFS and OS [21]. A longer PFS may improve the 2-year survival rate by decreasing the risk of death.

We observed that patients with tumors involving only a single lobe of the brain exhibited longer PFS than patients with multi-lobe involvement. A possible explanation is that GBM aggressively invades surrounding tissues, and invisible tumors can be more easily removed in the patients with tumors involving a single lobe of the brain. Similar to our study, Filippini, G. et al. conducted a survival analysis of 676 GBM patients and found that the prognosis of patients with single lobe involvement was significantly better than those with multiple lobe involvement (HR: 0.78, 95% CI (0.65–0.94), *P* = 0.008) [22]. In a study by Kaisorn, L et al., RV was closely related to tumor recurrence. In addition to being negatively related to the degree of resection, RV was also affected by tumor location. When tumors involve multiple lobes and may not be fully removed by surgery, long-term adjuvant TMZ chemotherapy can be of benefit to these patients [23].

Despite our interesting results, our study has several limitations. First, the overall sample size of patients included in the study was small and they all came from the same clinical center. Second, this is a non-randomized retrospective study and that differences in treatment selection after tumor recurrence may affect OS. Therefore, a prospective multicenter clinical trial is necessary to evaluate the question of duration of TMZ therapy better.

The Mini-Mental state examination (MMSE) is a simple test that is able to briefly estimate the cognitive status of a patient affected by a cognitive impairment either induced by a tumour, in other studies, MMSE has proven to be very useful to describe the tumor-related cognitive impairment [24]. Post-hoc analysis of neurocognitive functioning in the first year. Patients had an improved postoperative MMSE scores and KPS scores in both group compared with preoperative patients, indicating that aggressive treatment helped improve quality*-*of*-*life*.* Neurocognitive function decreased in both groups at T4, but group B was significantly higher than group A, which was associated with the differences of tumor progression between the two groups (15/27 progressions in group A, 1/26 progressions in group B).

In conclusion, long-term adjuvant TMZ chemotherapy was beneficial for PFS and 2-year survival rate in GBM patients, and improved their quality of life contemporarily. But OS was not significantly improved.

## Data Availability

The datasets used and/or analyzed during the current study are available from the corresponding author on reasonable request.

## Abbreviations

GBM: Glioblalstoma
PFS: Progression-free Survival
CNS: Central Nervous System
TMZ: Temozolomide
OS: Overa survive
MGMT: 0–6-Methyguanine DNA
IDH: Isocitrate dehydrogenase
KPS: Karnofsky
RT: Radiotherapy
OR: Odds ratio
RV: Residual volume
